# Secosteroids and Norcembranoids from the Soft Coral *Sinularia nanolobata*

**DOI:** 10.3390/md11093288

**Published:** 2013-08-27

**Authors:** Yen-Ju Tseng, Shang-Kwei Wang, Chang-Yih Duh

**Affiliations:** 1 Department of Marine Biotechnology and Resources, National Sun Yat-sen University, Kaohsiung 804, Taiwan; E-Mail: pit0424@yahoo.com.tw; 2 Department of Microbiology, Kaohsiung Medical University, Kaohsiung 807, Taiwan; 3 Asia-Pacific Ocean Research Center, National Sun Yat-sen University, Kaohsiung 804, Taiwan

**Keywords:** soft coral, *Sinularia nanolobata*, secosteroid, cytotoxicity

## Abstract

Two new 9,11-secosteroids, 22α-acetoxy-24-methylene-3β,6α,11-trihydroxy-9,11-seco-cholest-7-en-9-one (**1**) and 11-acetoxy-24-methylene-1β,3β,6α-trihydroxy-9,11-seco-cholest-7-en-9-one (**2**), as well as two known norcembranoids, 5-*epi*-sinuleptolide (**3**) and sinuleptolide (**4**), were isolated from the soft coral *Sinularia nanolobata*. The structures of these metabolites were elucidated on the basis of extensive spectroscopic analysis. The anti-HCMV (human cytomegalovirus) activity of **1**–**4** and its cytotoxicity against selected cell lines were evaluated.

## 1. Introduction

**Figure 1:**
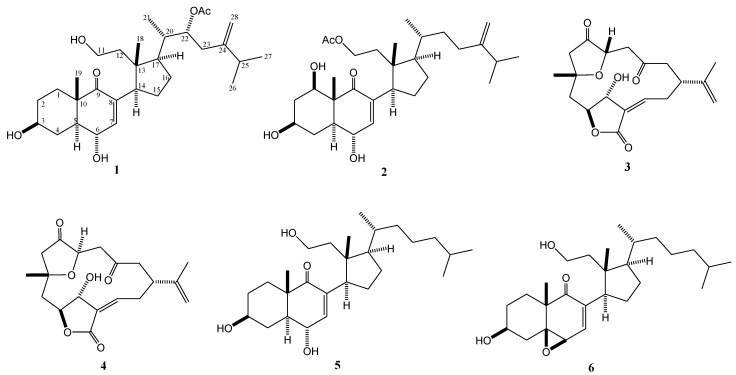
Structures of Metabolites **1**–**6**.

Soft corals have been proven to be a rich source of 9,11-secosterols [1,2,3,4,5,6,7,8,9,10,11,12,13,14] and C-4 norcembranoids [15,16,17,18,19,20,21,22,23,24]. 9,11-Secosteroids were found to possess cytotoxic [2,3,5,6,8,10,11,12,13] and anti-inflammatory activities [13]. C-4 Norcembranoids were found to possess cytotoxic [18,19,20,25], anti-inflammatory [23,24], and antiviral activities [23]. As part of the continuing search for bioactive metabolites from marine invertebrates, we explored the secondary metabolites of the soft coral *Sinularia nanolobata* (Verseveldt, 1977) which was collected from Xiao-Liuqiu Island, Pingtung County, Taiwan. Chromatographic separation of the EtOAc extract of the soft coral resulted in the isolation of new secosteroids **1** and **2**, along with two known norcembranoids, 5-*epi*-sinuleptolide (**3**) and sinuleptolide (**4**) [21] (Figure 1). The structures of these metabolites were established by extensive spectroscopic analysis. The anti-HCMV (human cytomegalovirus) activity of **1**–**4** and cytotoxicity against P-388 (mouse lymphocytic leukemia), HT-29 (human colon adenocarcinoma), and A-549 (human lung carcinoma) cancer cell lines were evaluated *in vitro*.

## 2. Results and Discussion

**Figure 2:**
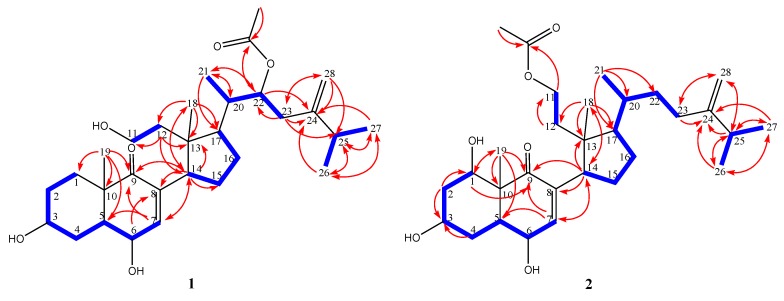
Selected ^1^H−^1^H COSY (▬) and HMBC (**→**) correlations of **1** and **2**.

Metabolite **1** was isolated as colorless needles. Its molecular formula, C_30_H_48_O_6_, was established by HRESIMS (*m/z* 527.3351, [M + Na]^+^), implying seven degrees of unsaturation. The presence of hydroxyl groups was suggested by a strong absorption band at 3459 cm^−1^ in the IR spectrum. The ^13^C NMR and DEPT (Table 1) spectroscopic data showed signals of six methyls, eight sp^3^ methylenes (including one oxymethylene), one sp^2^ methylene, eight sp^3^ methines (including three oxymethines), one sp^2^ methine, two sp^3^ and four sp^2^ quaternary carbons. The quaternary carbon signal at δ_C_ 205.4 combined with the chemical shifts of H_3_-18 (δ 0.67, s), H_3_-19 (δ 1.14, s), H_3_-21 (δ 1.02, d, *J* = 6.8 Hz), H_3_-26 (δ 1.03, d, *J* = 6.8 Hz), H_3_-27 (δ 1.03, d, *J* = 6.8 Hz), H-3 (δ 3.61, m), H-6 (δ 4.30, dd, *J* = 10.0, 1.6 Hz), H-7 (δ 6.61, d, *J* = 1.6 Hz), and H_2_-11 (each 1H, δ 3.70, m; δ 3.93, m) were found to be similar to those of 3β,6α,11-trihydroxy-9,11-seco-5α-cholest-7-en-9-one (**5**) [4]. Moreover, the signals appearing at δ_C_ 170.7 (qC), 152.2 (qC), 108.8 (CH_2_), 74.7 (CH), and 21.2 (CH_3_) indicated the presence of an acetoxy and an exomethylene in **1**. This was further supported by the ^1^H NMR signals of acetoxy and exomethylene protons at δ_H_ 1.99 (3H, s) and 4.71 and 4.80 (each 1H, s), respectively. From the COSY spectrum measured in CDCl_3_, it was possible to establish five proton sequences from H-1 to H-7, H_2_-11 to H_2_-12, H-14 to H-17, H-21 to H_2_-23 through H-20, and H-25 to H_3_-26 and H_3_-27 (Figure 2). Key HMBC correlations of H_2_-4 to C-3 and C-5; H-7 to C-5, C-9 and C-14; H_2_-12 to C-13 and C-14; H-14 to C-7, C-8, C-9, C-13, C-15 and C-18; H_3_-18 to C-12, C-13, C-14, and C-17; H_3_-19 to C-1, C-5, C-9, and C-10; H-20 to C-21; H_3_-21 to C-17, C-20 and C-22; H-22 to C-24; H_2_-23 to C-25; H_3_-26 to C-24, C-25, and C-27; H_3_-27 to C-24, C-25, and C-26; H_2_-28 to C-23, C-24 and C-25; H_3_-OAc to OAc-22 permitted the establishment of the secosterol-type skeleton of **1**.

**Table 1:** ^1^H and ^13^C NMR Spectroscopic Data for compounds **1** and **2**.

Position	1		2	
	δ_H_ *^a^*	δ_C_ *^b^*	δ_H_ *^a^*	δ_C_ *^b^*
1	1.48 m; 1.90 m	31.8, CH_2_ *^d^*	3.87 dd (12.0, 4.4)	70.7, CH
2	1.48 m; 1.94 m	30.5, CH_2_	1.52 m; 2.14 m	37.8, CH_2_
3	3.61 m	69.9, CH	3.71 m	67.4, CH
4	1.45 m; 2.31 m	32.8, CH_2_	1.43 m; 2.29 m	32.2, CH_2_
5	1.80 m	48.5, CH	1.70 m	46.4, CH
6	4.30 dd (10.0, 1.6) *^c^*	69.4, CH	4.38 dd (9.2, 2.0)	69.1, CH
7	6.61 d (1.6)	147.8, CH	6.58 d (1.6)	147.4, CH
8		136.5, qC		136.8, qC
9		205.4, qC		206.5, qC
10		44.9, qC		49.0, qC
11	3.70 m; 3.93 m	59.3, CH_2_	4.16 td (10.4, 6.4); 4.23 td (10.4, 5.2)	61.2, CH_2_
12	1.04 m; 1.64 m	40.7, CH_2_	1.15 m; 1.70 m	36.8, CH_2_
13		46.3, qC		46.0, qC
14	3.47 dd (9.6, 8.8)	42.2, CH	3.25 dd (10.8, 9.6)	42.2, CH
15	1.62 m	26.4, CH_2_	1.62 m	26.4, CH_2_
16	1.65 m; 1.91 m	27.0, CH_2_	1.45 m; 1.87 m	26.2, CH_2_
17	1.75 m	45.9, CH	1.67 m	50.1, CH
18	0.67 s	16.9, CH_3_	0.69 s	17.0, CH_3_
19	1.14 s	16.2, CH_3_	1.17 s	9.8, CH_3_
20	1.78 m	39.8, CH	1.46 m	34.7, CH
21	1.02 d (6.8)	11.8, CH_3_	1.02 d (6.4)	18.8, CH_3_
22	5.12 m	74.7, CH	1.18 m; 1.56 m	34.0, CH_2_
23	2.17 m	32.6, CH_2_	1.89 m; 2.11 m	31.6, CH_2_
24		152.2, qC		156.5, qC
25	2.20 m	33.4, CH	2.21 m	33.8, CH
26	1.03 d (6.8)	21.6, CH_3_	1.02 d (6.8)	21.8, CH_3_
27	1.03 d (6.8)	22.1, CH_3_	1.02 d (6.8)	22.0, CH_3_
28	4.71 s; 4.80 s	108.8, CH_2_	4.66 s; 4.73 s	106.2, CH_2_
OAc	1.99 s	21.2, CH_3_ 170.7, qC	2.01 s	21.1 CH_3_ 171.2, qC

*^a^* Spectra recorded at 400 MHz in CDCl_3_; *^b^* Spectra recorded at 100 MHz in CDCl_3_; *^c^ J* values (in Hz) are in parentheses; *^d^* Carbon types are deduced by HSQC and DEPT experiments.

The relative configurations of eight chiral centers at C-3, C-5, C-6, C-10, C-13, C-14, C-17, and C-20 in **1** were found to be the same as those of 3β,6α,11-trihydroxy-9,11-seco-5α-cholest-7-ene-9-one [4] (Figure 2). Key NOE correlations for **1** showed interactions between H-20/H-22. Thus, OAc-22 should be α-oriented. The above data established the structure of compound **1** as 22*R*-acetoxy-3β,6α,11-trihydroxy-24-methylene-9,11-secocholestan-9-one.

**Figure 3:**
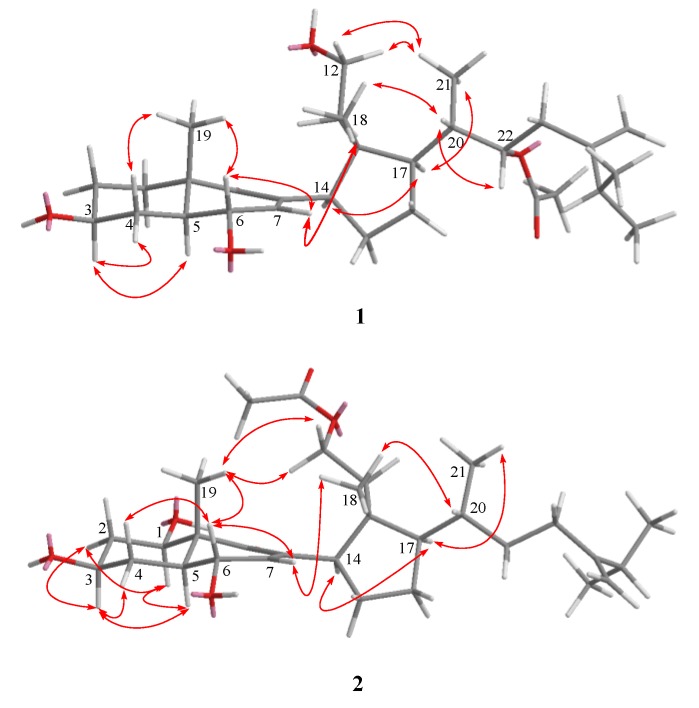
Key NOESY Correlations for **1** and **2**.

Metabolite **2** possessed the same molecular formula (C_30_H_48_O_6_) as that of **1**, as revealed from HRESIMS. The ^13^C NMR spectral data of **2** were found to be close to those of **1**, except for the signals due to C-1, C-11, and C-22. COSY correlations from H-1 to H-2, H_2_-11 to H_2_-12, and H-20 to H_2_-22. as well as HMBC correlations from H-1 to C-10, C-9, from H_2_-11 to 11-OAc, and from Me-21 to C-17, C-20, C-22 confirmed the presence of a hydroxyl at C-1, an acetoxyl at C-11, and the absence of the acetoxyl at C-22 (Figure 2). Furthermore, the configuration of C-1 was deduced from the NOE correlations of H-1/H-5 and H-3/H-5 (Figure 3). Thus, the structure of compound **2** was established as 11-acetoxy-24-methylene-1β,3β,6α-trihydroxy-9,11-seco-cholest-7en-9-one.

Cytotoxicity of compounds **1**–**4** against the proliferation of a limited panel of cancer cell lines, including P-388, A549, and HT-29, were evaluated (Table 2). Metabolites **1**–**3** displayed weak cytotoxicity against P-388, with IC_50_ of 10.2, 27.8, and 15.7 μg/mL, respectively. Compounds **1**–**4** was also examined for antiviral activity against human cytomegalovirus (HCMV) using a human embryonic lung (HEL) cell line. Compounds **4** showed anti-HCMV activity with ED_50_ of 1.92 μg/mL (Ganciclovir showed anti-HCMV activity with ED_50_ of 0.12 μg/mL).

**Table 2:** Cytotoxicity and anti- human cytomegalovirus (HCMV) activity of **1**–**4**.

Compounds	IC_50_ (μg/mL)				Anti-HCMV
	A549	HT-29	P-388	HEL	
**1**	>50	>50	10.2	>50	>50
**2**	>50	>50	27.8	>50	>50
**3**	>50	>50	15.7	>50	>50
**4**	>50	>50	>50	>50	1.92

## 3. Experimental Section

### 3.1. General Experimental Procedures

Optical rotations were measured on a JASCO P1020 digital polarimeter. IR spectra were recorded on a JASCO FT/IR4100 infrared spectrophotometer. The NMR spectra were recorded on a Varian MR 400 FT-NMR at 400 MHz for ^1^H and 100 MHz for ^13^C, in CDCl_3_ using solvent peak as internal standard. LRMS and HRMS were obtained by ESI on a Bruker APEX ΙΙ mass spectrometer. Silica gel 60 (Merck, Darmstadt, Germany, 230–400 mesh) and LiChroprep RP-18 (Merck, 40–63 μm) were used for column chromatography. Precoated silica gel plates (Merck, Kieselgel 60 F_254_, 0.25 mm) and precoated RP-18 F_254s_ plates (Merck) were used for TLC analysis. High-performance liquid chromatography was carried out using a Hitachi L-7100 pump equipped with a Hitachi L-7400 UV detector at 220 nm together with a semi-preparative reversed-phase column (Merck, Hibar LiChrospher RP-18e, 5 μm, 250 × 25 mm).

### 3.2. Animal Material

The octocoral *S. nanolobata* was collected by hand using scuba at the Xiao Liuqiu, Pingtung County, Taiwan, in November 2011, at a depth of 6 m. This soft coral was identified by Prof. Chang-Fong Dai, Institute of Oceanography, National Taiwan University. A voucher specimen (SL-10) was deposited in the Department of Marine Biotechnology and Resources, National Sun Yat-sen University.

### 3.3. Extraction and Separation

The frozen soft coral (1.5 kg) was chopped into small pieces and extracted with EtOAc (3L × 3) in a percolator at room temperature. The EtOAc extract of *S. nanolobata* was concentrated to a dark brown gum (12.3 g). The EtOAc residue was subjected to Si 60 CC using *n*-hexane-EtOAc-MeOH mixtures of increasing polarity for elution. Fraction 15~21, eluted with *n*-hexane-EtOAc (1:10) to EtOAc-MeOH (2:1), was further subjected to Si 60 CC EtOAc-MeOH (5:1) to give four subfractions. A subfraction 15~21-1, was purified by reverse-phase HPLC (MeOH-H_2_O, 55:45) to afford **3** (50.0 mg) and **4** (35.0 mg). A subfraction 15~21-4, was purified by reverse-phase HPLC (MeOH-H_2_O, 85:15) to afford **1** (2.5 mg) and **2** (3.1 mg).

22α-acetoxy-24-methylene-3β,6α,11-trihydroxy-9,11-seco-cholest-7-en-9-one (**1**): White powder; [α]^25^_D_ = +54 (*c* 0.6, CHCl_3_); IR (KBr) ν_max_ 3459, 2956, 1733, 1031, 1009 cm^−1^; ^1^H and ^13^C NMR data, see Table 1; ESIMS *m*/*z* 527 [M + Na]^+^; HRESIMS *m*/*z* 527.3351 (calcd for C_30_H_48_O_6_Na, 527.3348).

11-acetoxy-24-methylene-1β,3β,6α-trihydroxy-9,11-seco-cholest-7-en-9-one (**2**): White powder; [α]^25^_D_ = +16 (*c* 0.8, CHCl_3_); IR (KBr) ν_max_ 3444, 2925, 1741, 1260, 1036 cm^−1^; ^1^H and ^13^C NMR data, see Table 1; ESIMS *m*/*z* 527 [M + Na]^+^; HRESIMS *m*/*z* 527.3345 (calcd for C_30_H_48_O_6_Na, 527.3348).

### 3.4. Cytotoxicity Testing

Cytotoxicity was determined on P-388 (mouse lymphocytic leukemia), HT-29 (human colon adenocarcinoma), and A-549 (human lung epithelial carcinoma) tumor cells using a modification of the MTT colorimetric method according to a previously described procedure [26,27]. The provision of the P-388 cell line was supported by J.M. Pezzuto, formerly of the Department of Medicinal Chemistry and Pharmacognosy, University of Illinois at Chicago. HT-29 and A-549 cell lines were purchased from the American Type Culture Collection. To measure the cytotoxic activities of tested compounds, five concentrations with three replications were performed on each cell line. Mithramycin was used as a positive control.

### 3.5. Anti-HCMV Assay

To determine the effects of natural products upon HCMV cytopathic effect (CPE), confluent human embryonic lung (HEL) cells grown in 24-well plates were incubated for 1 h in the presence or absence of various concentrations of tested natural products with three replications. Ganciclovir was used as a positive control. Then, cells were infected with HCMV at an input of 1000 pfu (plaque forming units) per well of a 24-well dish. Antiviral activity was expressed as IC_50_ (50% inhibitory concentration), or compound concentration required to reduce virus induced CPE by 50% after 7 days as compared with the untreated control. To monitor the cell growth upon treating with natural products, an MTT-colorimetric assay was employed [28].

## 4. Conclusions

In the previous studies, 5-*epi*-sinuleptolide (**3**) and sinuleptolide (**4**) both showed inhibition of iNOS protein by LPS stimulation [23], TNF-α production in a dose-dependent manner [24], and nitric oxide (NO) production [24]. 5-*Epi*-sinuleptolide (**3**) inhibited human skin cancer cells growth [25]. Sinuleptolide (**4**) exerted antiviral activity against HCMV (human cytomegalovirus) cells [23]. 9,11-Secosteroid **6** with epoxide at C-5 and C-6 exhibited cytotoxicity against HT-29 cells [29]. This investigation of soft coral *S. nanolobata* collected at Xiao-Liuqiu Island (Pingtung County, Taiwan) has led to the isolation of two new 9,11-secosteroids (**1** and **2**) and two known compounds, 5-*epi*-sinuleptolide (**3**) and sinuleptolide (**4**). Metabolites **1**–**3** displayed weak cytotoxicity and selectivity against P-388, with IC_50_ of 10.2, 27.8, and 15.7 μg/mL, respectively. Metabolite **4** exhibited antiviral activity against HCMV with ED_50_ of 1.92 μg/mL.

## Supplementary Files

Supplementary File 1 Supplementary Materials (PDF, 610 KB)
