# Supplementary material for: Secosteroids and Norcembranoids from the Soft Coral Sinularia nanolobata
Source: Mar Drugs. 2013 Aug 27;11(9):3288–96. doi: 10.3390/md11093288 (PMC3801121; doi:10.3390/md11093288)

Supplementary Materials

Table of Contents

Figure S1. ^{1}H NMR spectrum (400 MHz) of compound **1** in $CDCl_3$. 2

Figure S2. ^{13}C NMR spectrum (100 MHz) of compound **1** in $CDCl_3$. 3

Figure S3. DEPT spectrum (100 MHz) of compound **1** in $CDCl_3$. 4

Figure S4. COSY spectrum (400 MHz) of compound **1** in $CDCl_3$. 5

Figure S5. HSQC spectrum (400 MHz) of compound **1** in $CDCl_3$. 6

Figure S6. HMBC spectrum (400 MHz) of compound **1** in $CDCl_3$. 7

Figure S7. NOESY spectrum (400 MHz) of compound **1** in $CDCl_3$. 8

Figure S8. ^{1}H NMR spectrum (400 MHz) of compound **2** in $CDCl_3$. 9

Figure S9. ^{13}C NMR spectrum (400 MHz) of compound **2** in $CDCl_3$. 10

Figure S10. COSY spectrum (400 MHz) of compound **2** in $CDCl_3$. 11

Figure S11. HSQC spectrum (400 MHz) of compound **2** in $CDCl_3$. 12

Figure S12. HMBC spectrum (400 MHz) of compound **2** in $CDCl_3$. 13

Figure S13. NOESY spectrum (400 MHz) of compound **2** in $CDCl_3$. 14

Figure S1. ^{1}H NMR spectrum (400 MHz) of compound **1** in $CDCl_3$.

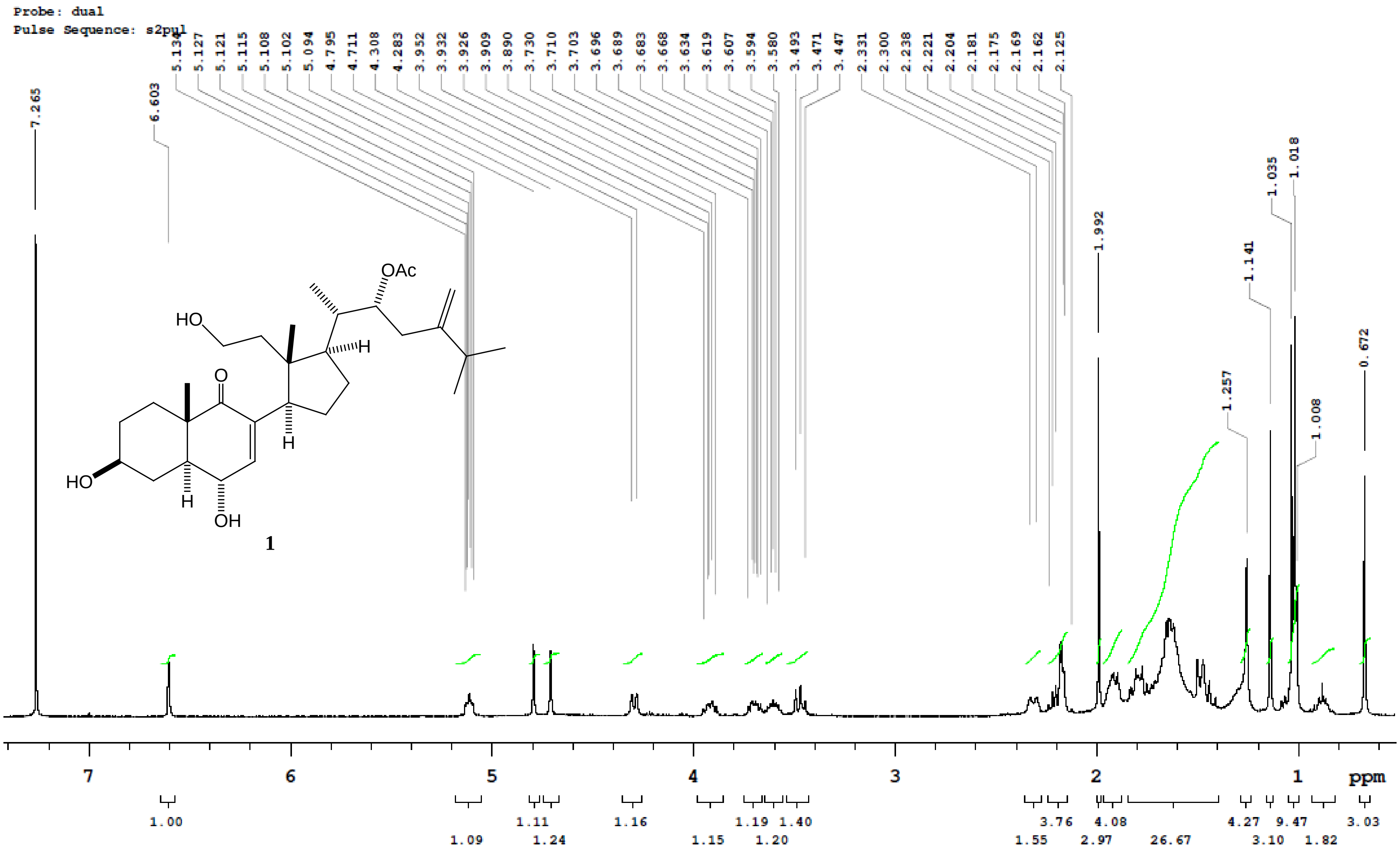

Figure S2. ^{13}C NMR spectrum (100 MHz) of compound **1** in $CDCl_3$.

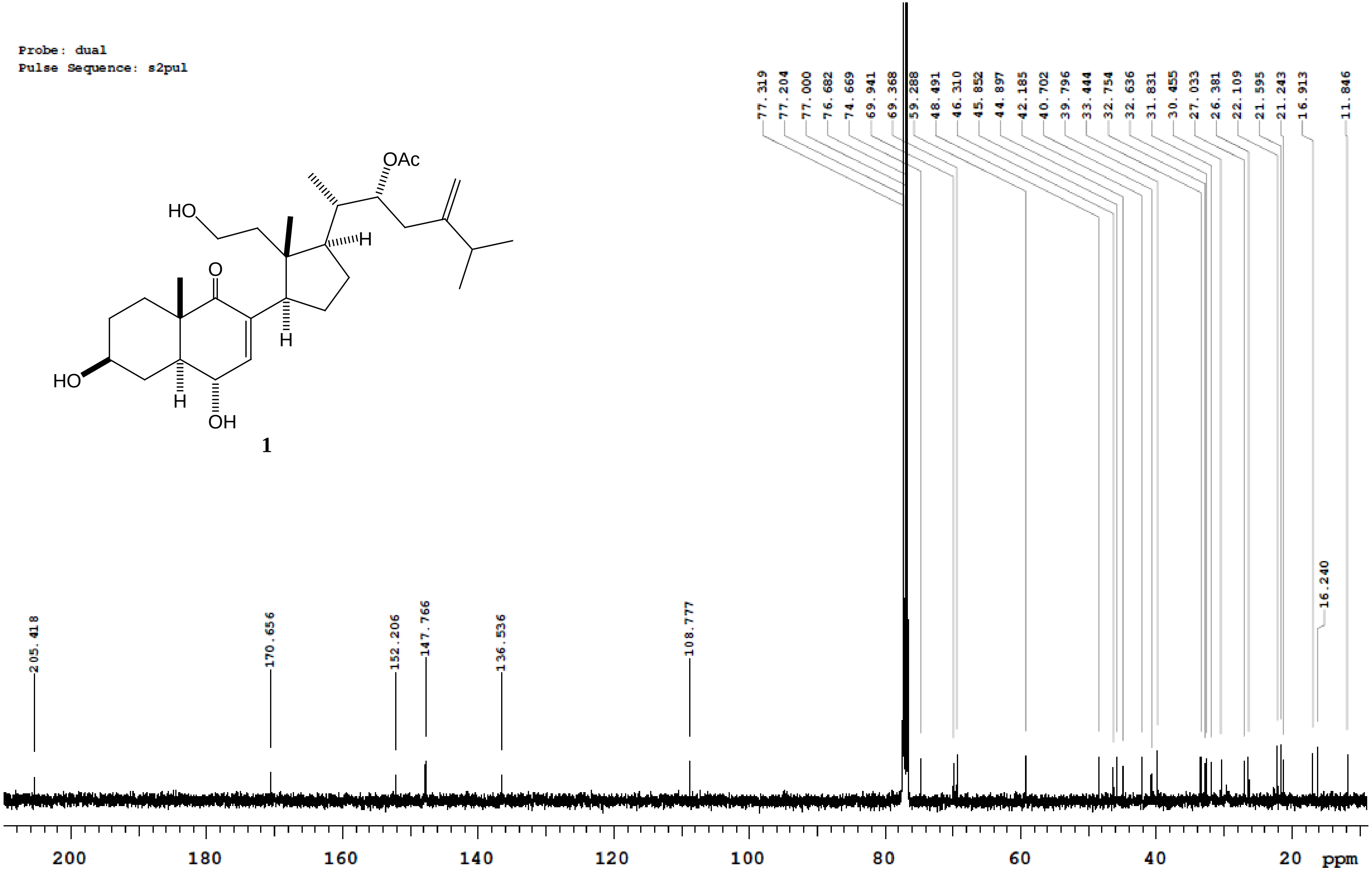

Figure S3. DEPT spectrum (400 MHz) of compound **1** in $CDCl_3$.

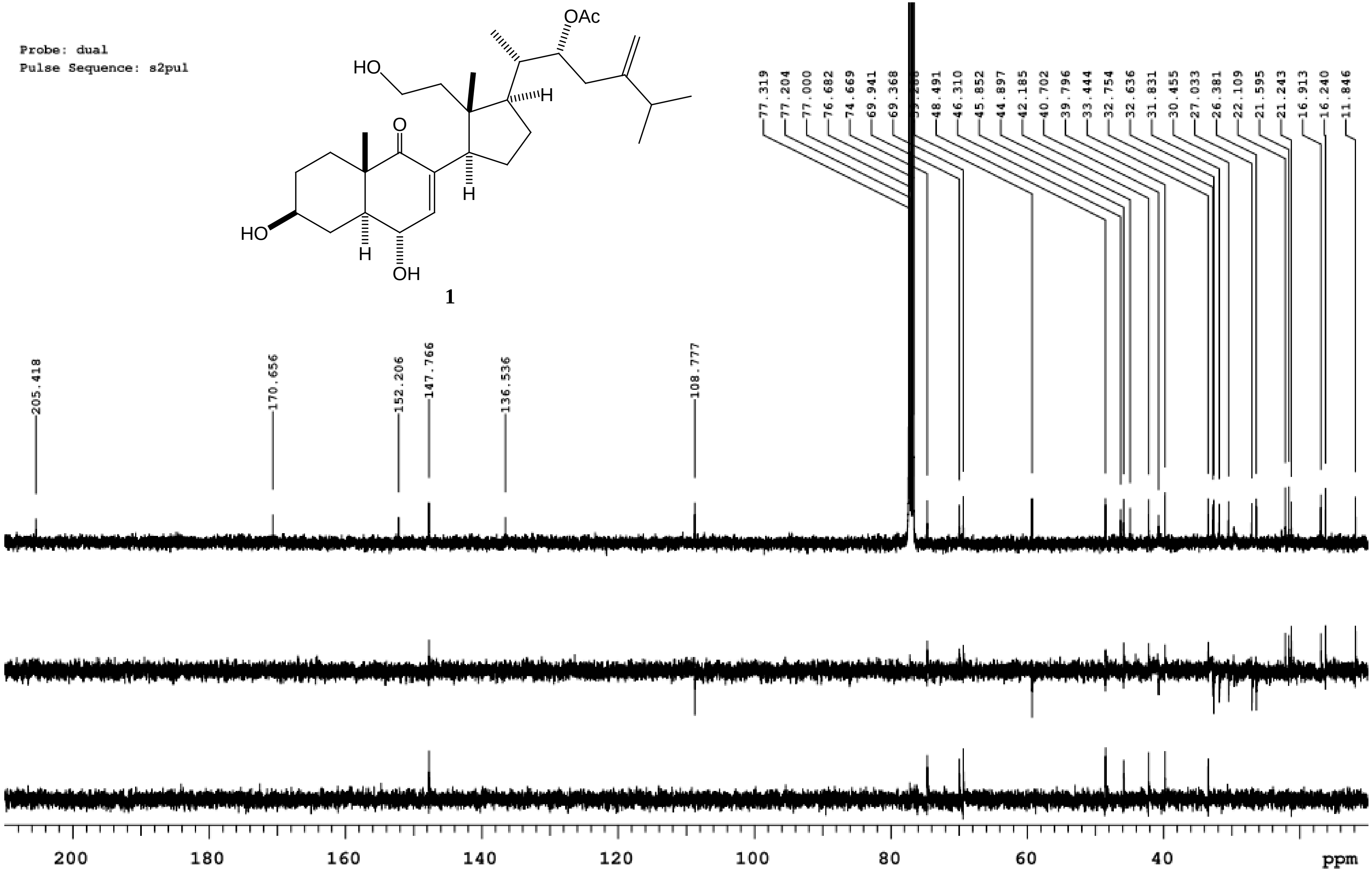

Figure S4. COSY spectrum (400 MHz) of compound **1** in $CDCl_3$.

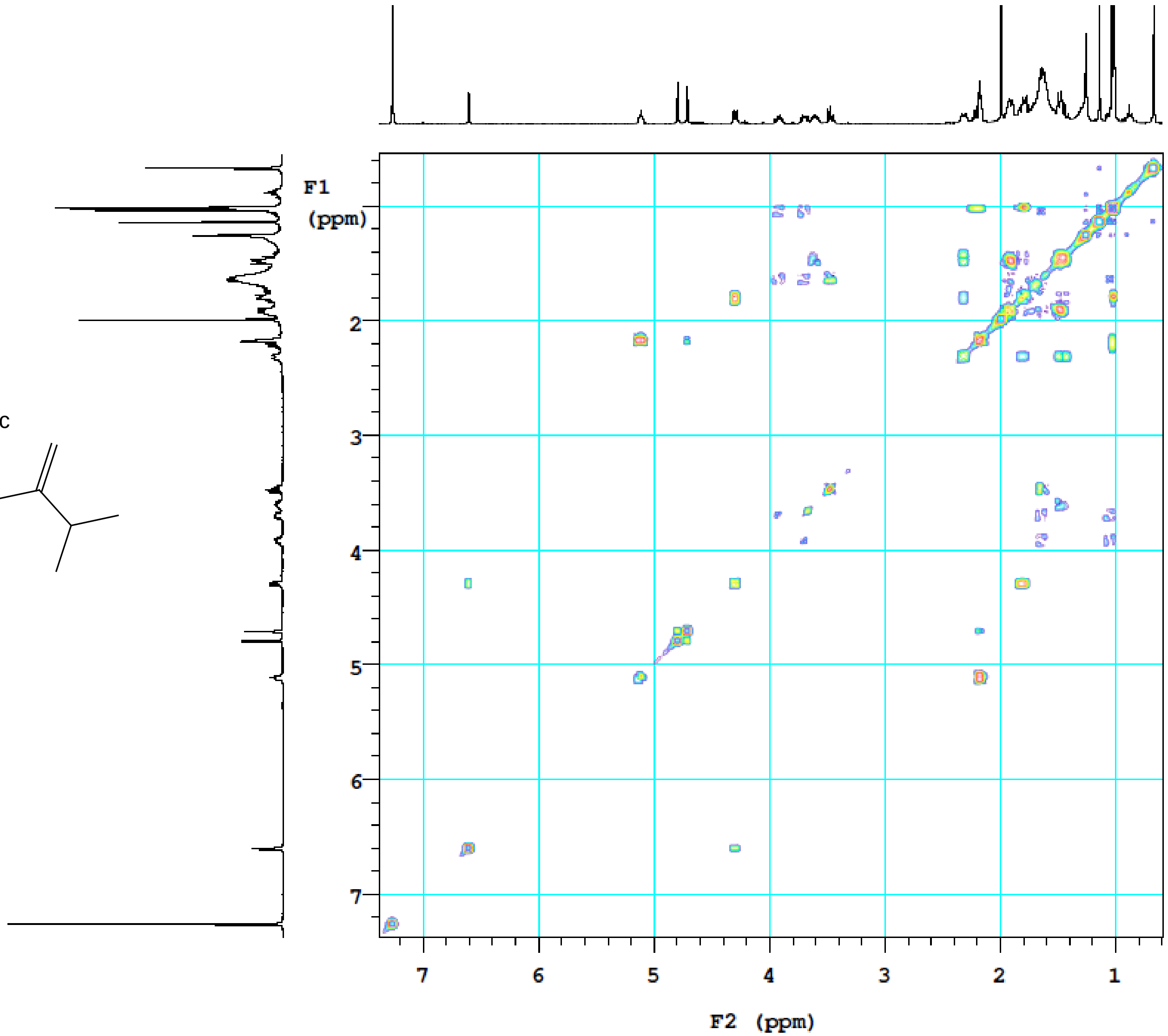

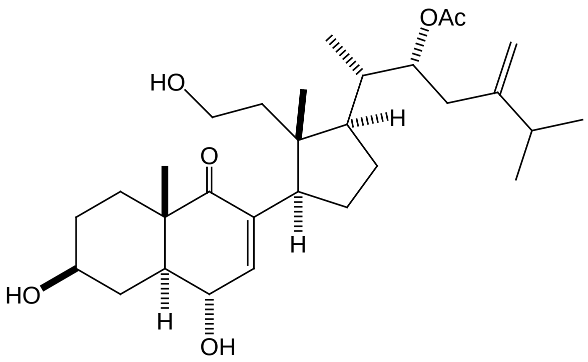

1

Figure S5. HSQC spectrum (400 MHz) of compound **1** in $CDCl_3$.

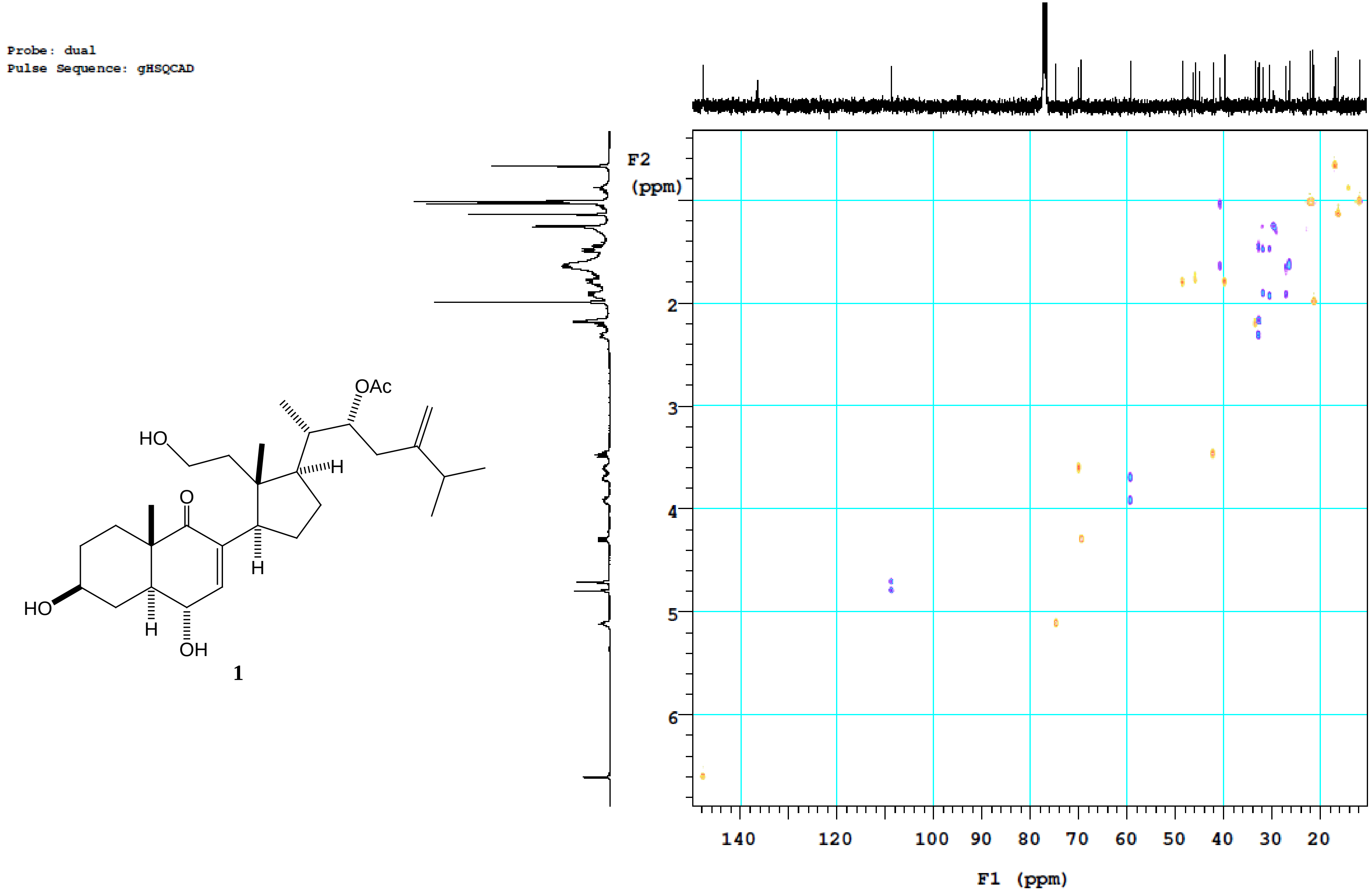

Figure S6. HMBC spectrum (400 MHz) of compound **1** in $CDCl_3$.

Probe: dual
Pulse Sequence: gHMBCAD

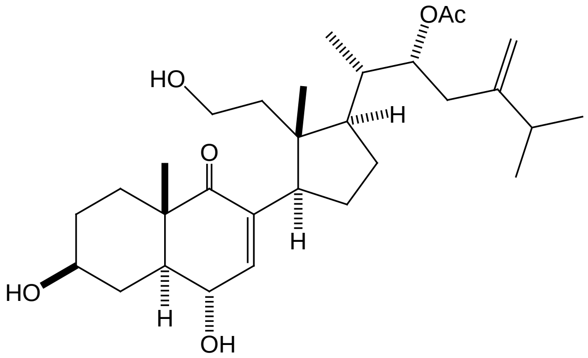

1

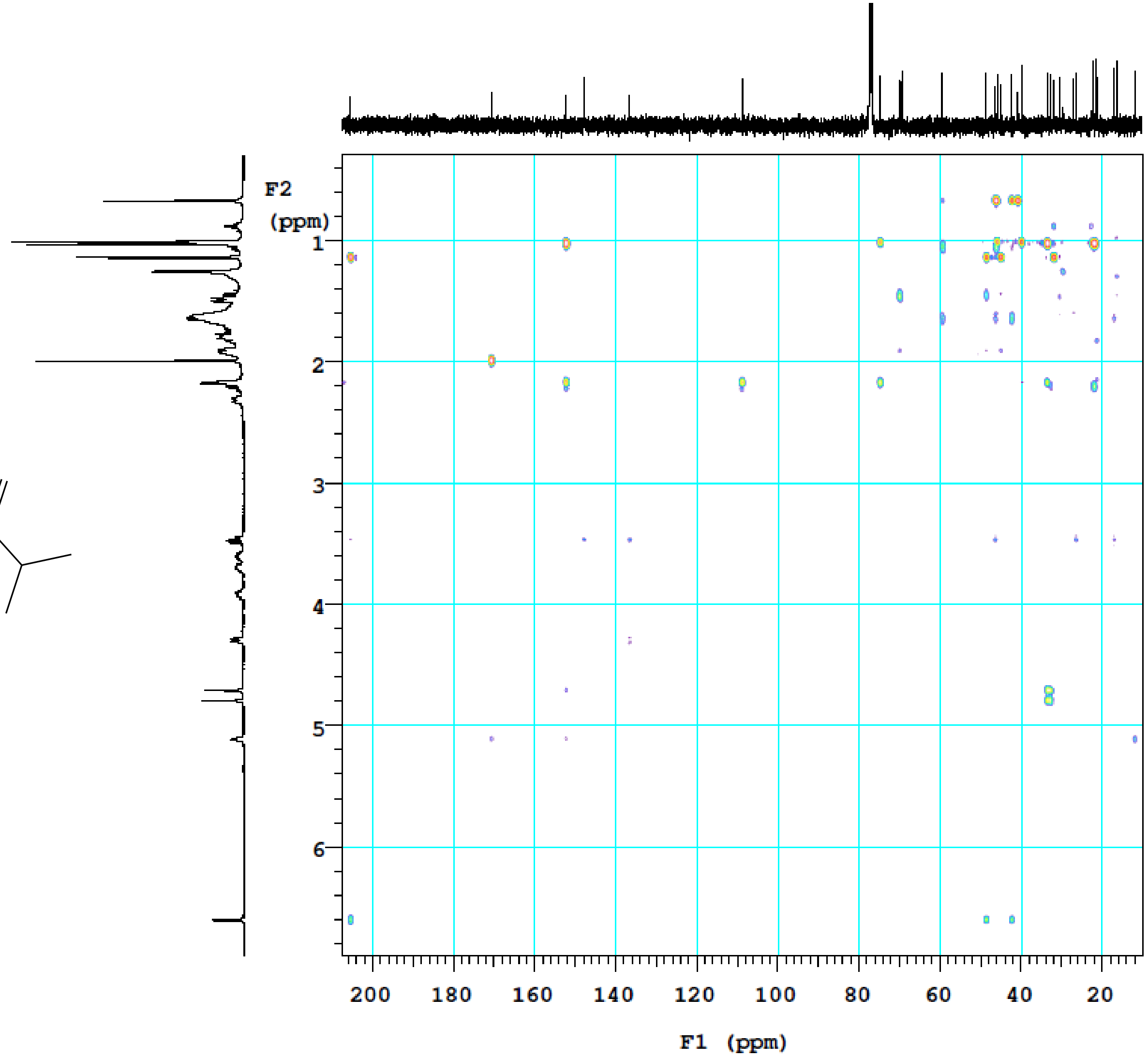

Figure S7. NOESY spectrum (400 MHz) of compound **1** in $CDCl_3$.

Probe: dual
Pulse Sequence: NOESY

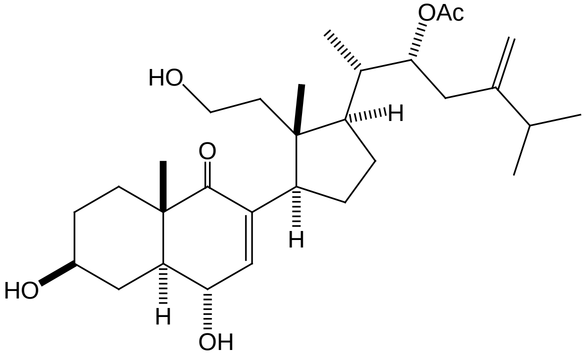

1

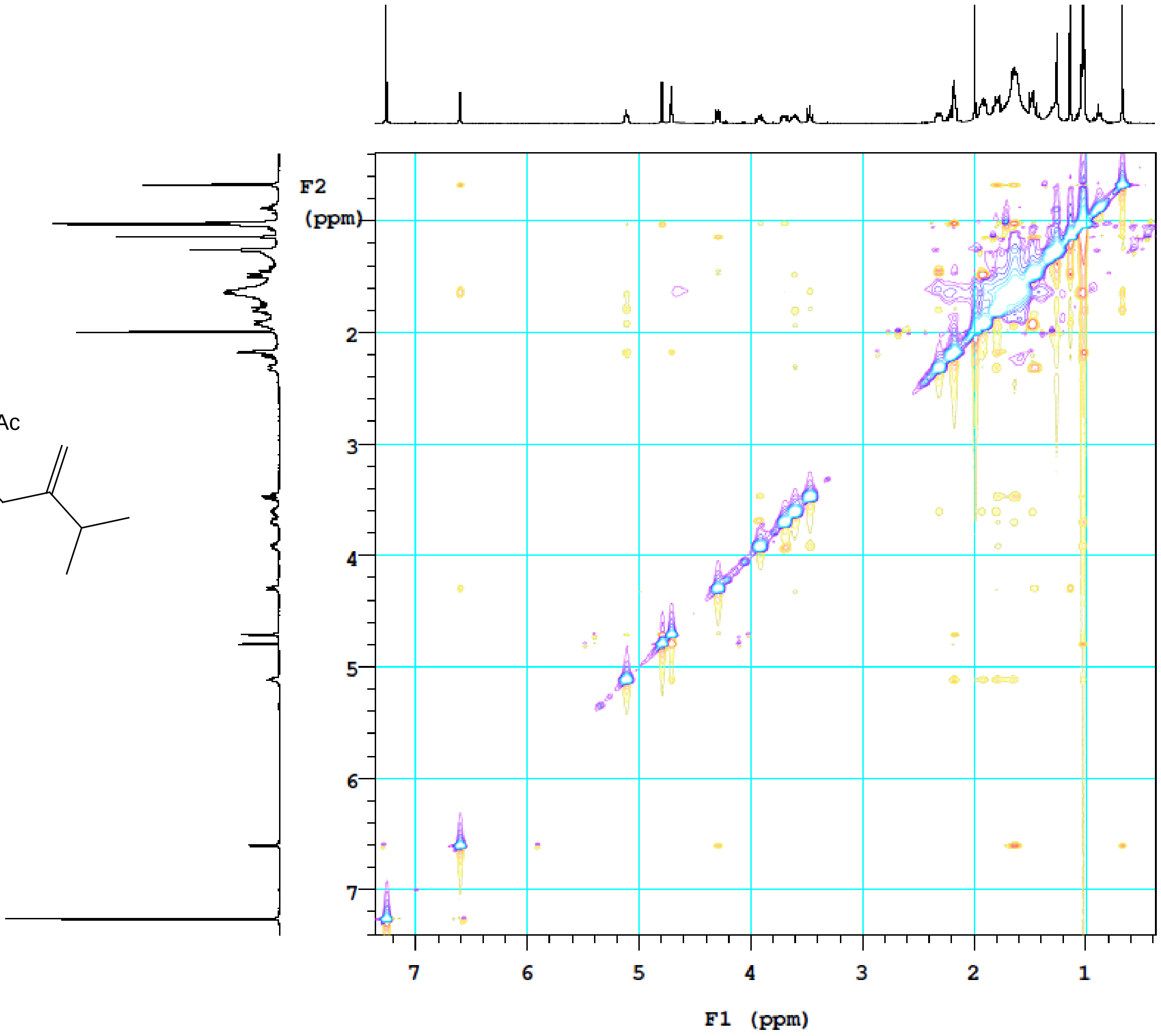

Figure S8. ^{1}H NMR spectrum (400 MHz) of compound **2** in $CDCl_3$.

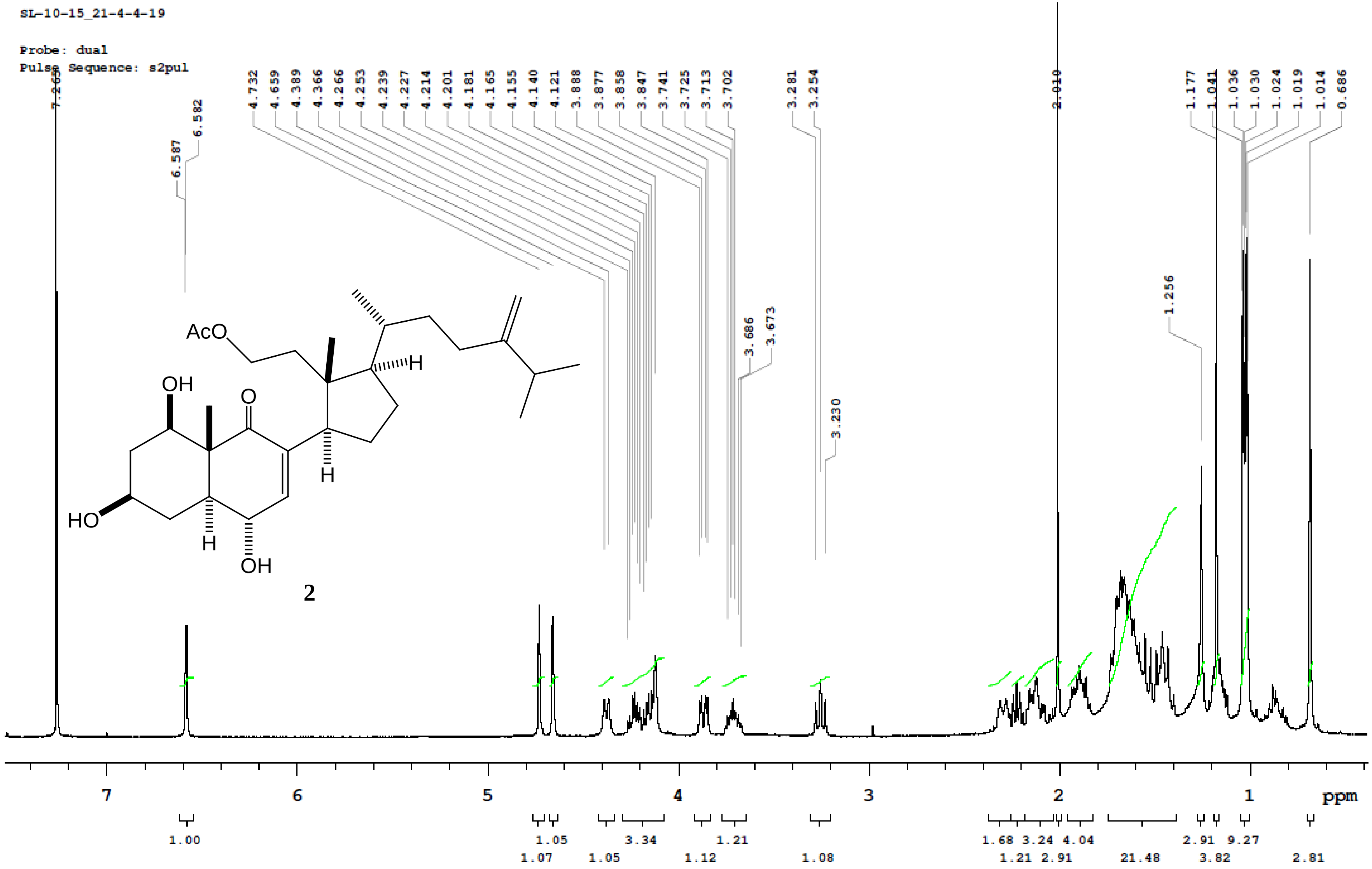

Mar. Drugs **2013**, *11*

Figure S9. ^{13}C NMR spectrum (100 MHz) of compound **2** in $CDCl_3$.

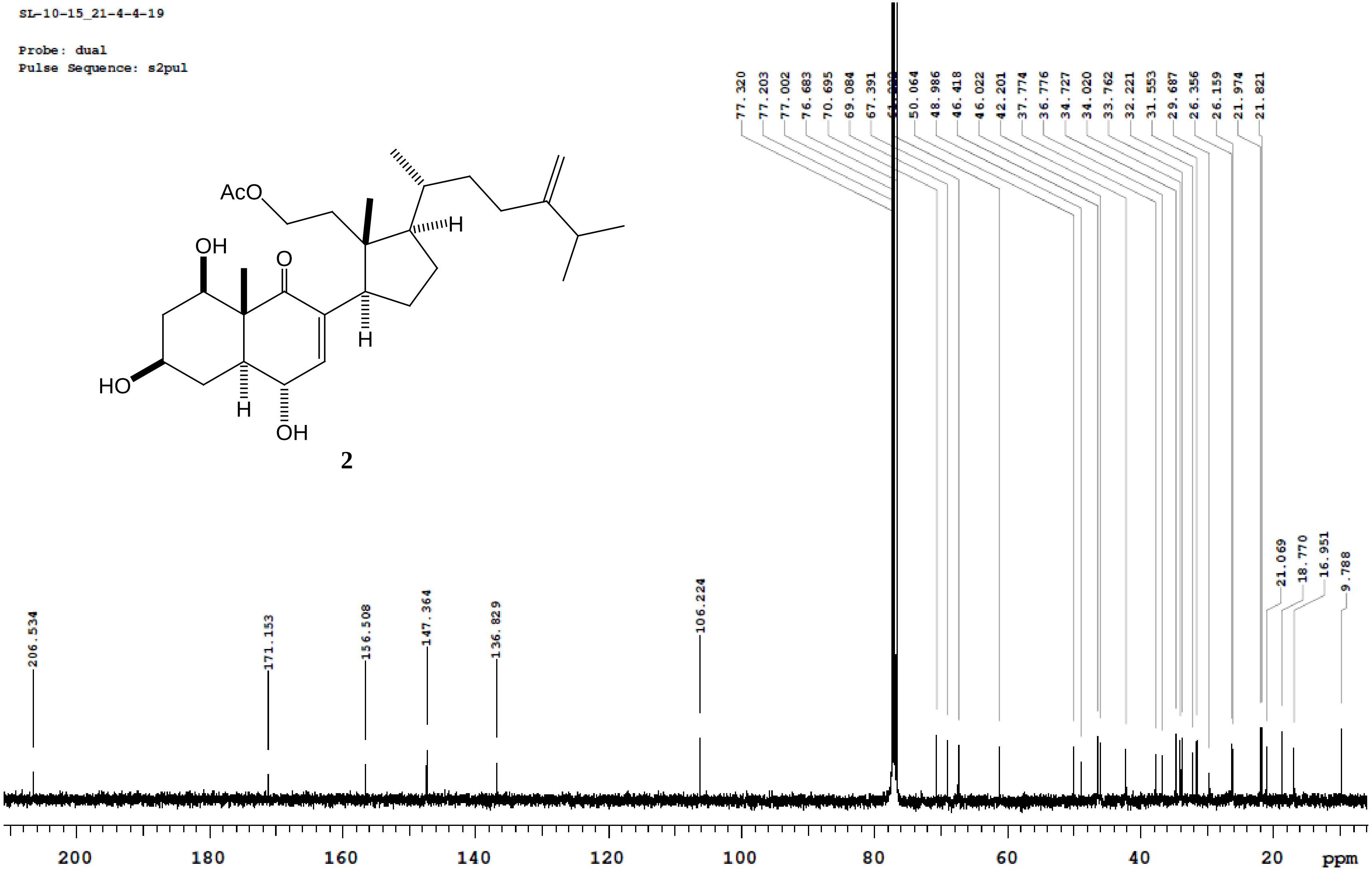

Figure S10. COSY spectrum (400 MHz) of compound **2** in $CDCl_3$.

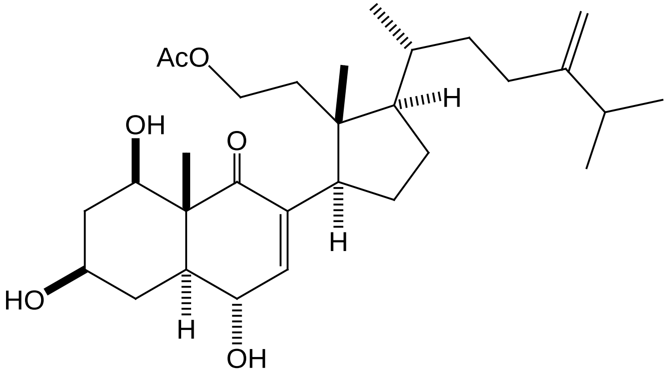

2

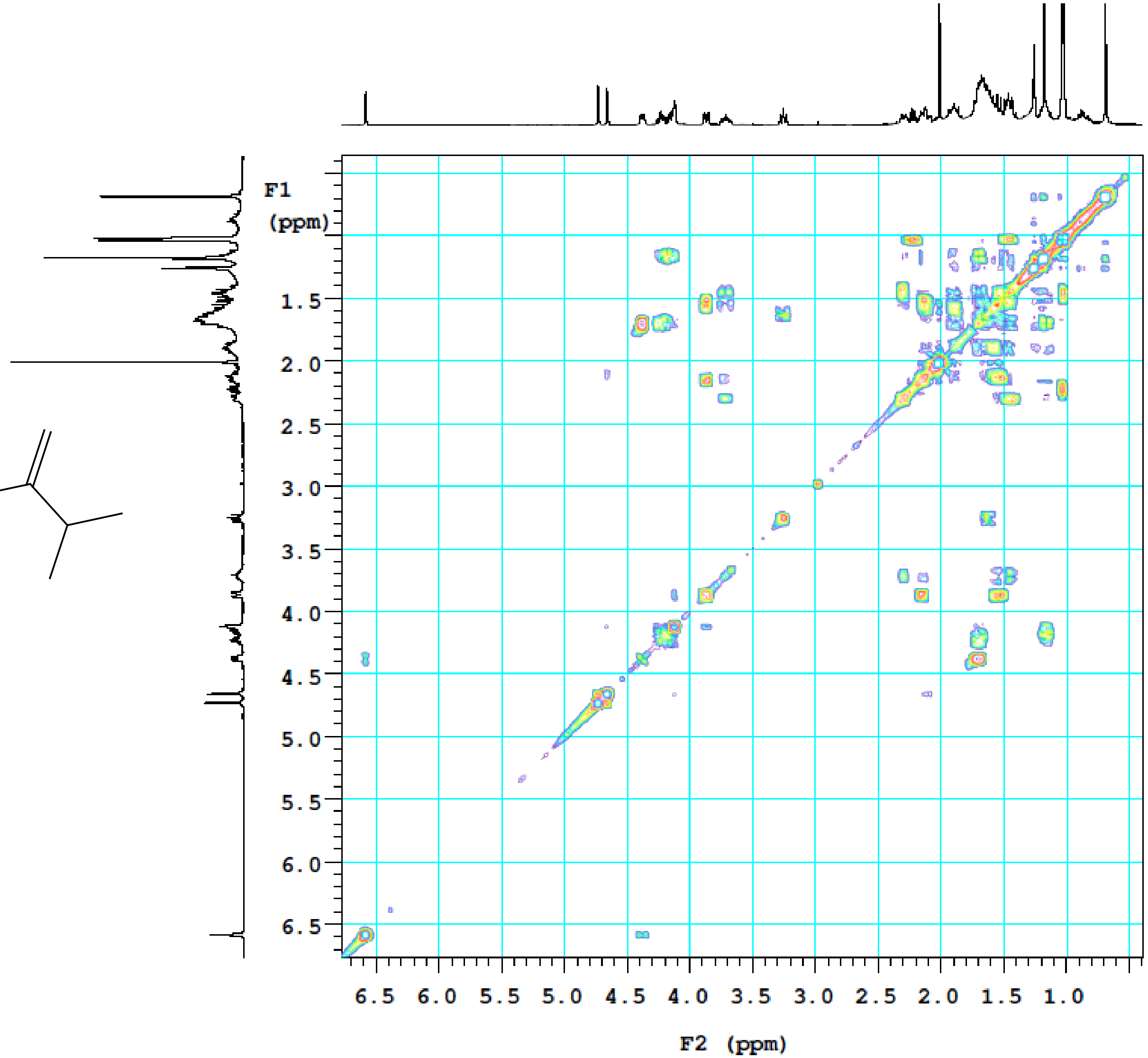

Figure S11. HSQC spectrum (400 MHz) of compound **2** in $CDCl_3$.

SL-10-15_21-4-4-19

Probe: dual
Pulse Sequence: gHSQCAD

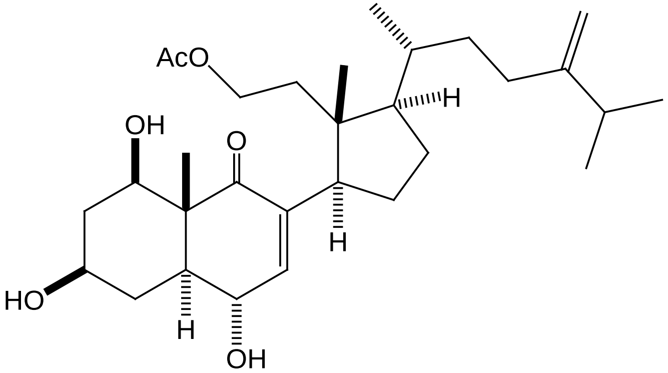

2

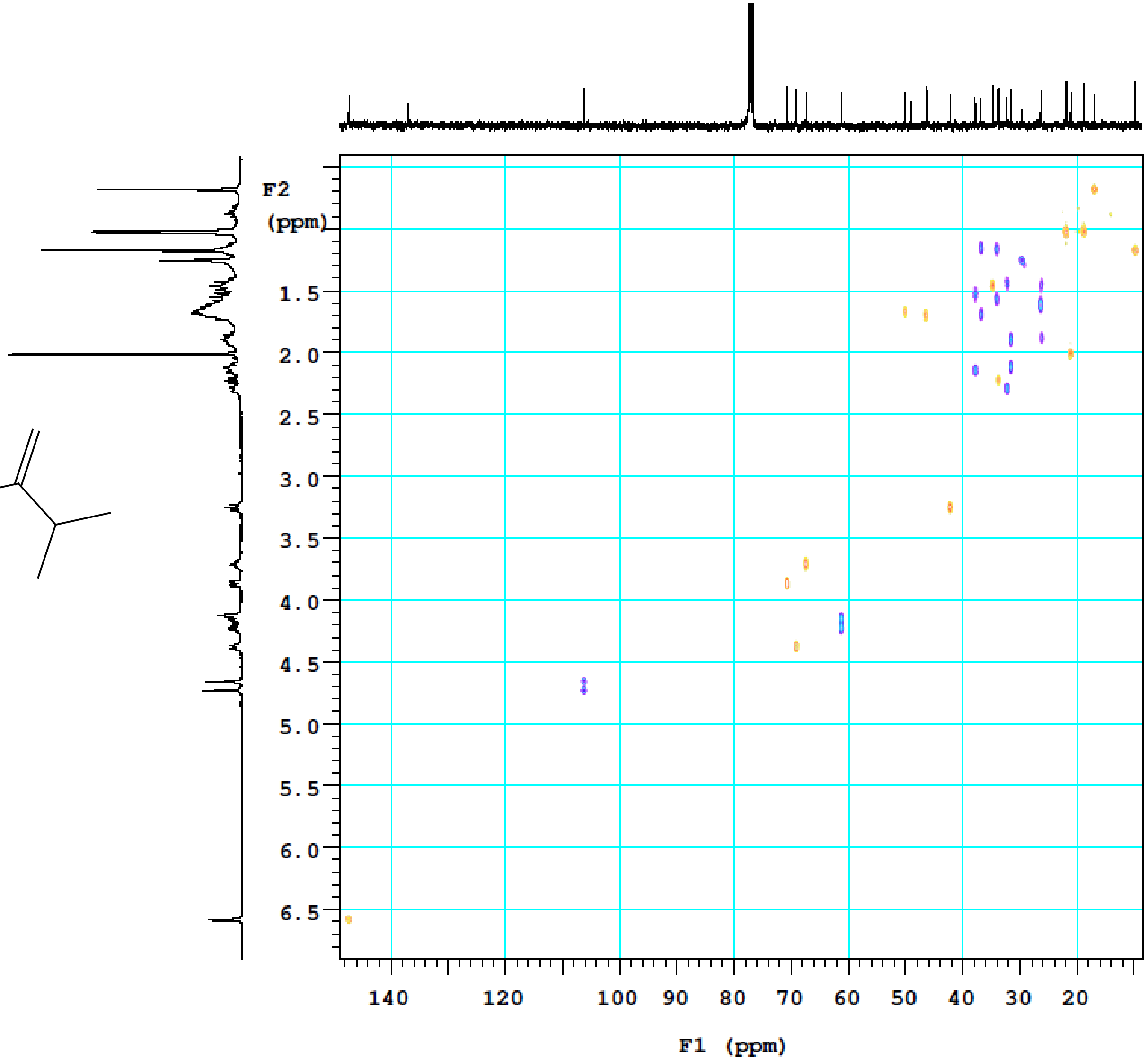

Figure S12. HMBC spectrum (400 MHz) of compound **2** in $CDCl_3$.

SL-10-15_21-4-4-19

Probe: dual
Pulse Sequence: gHMBCAD

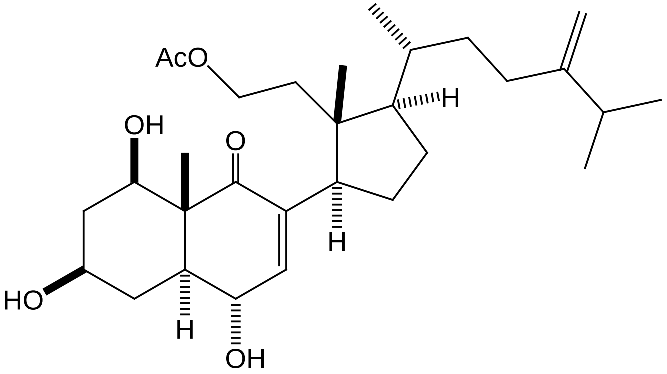

2

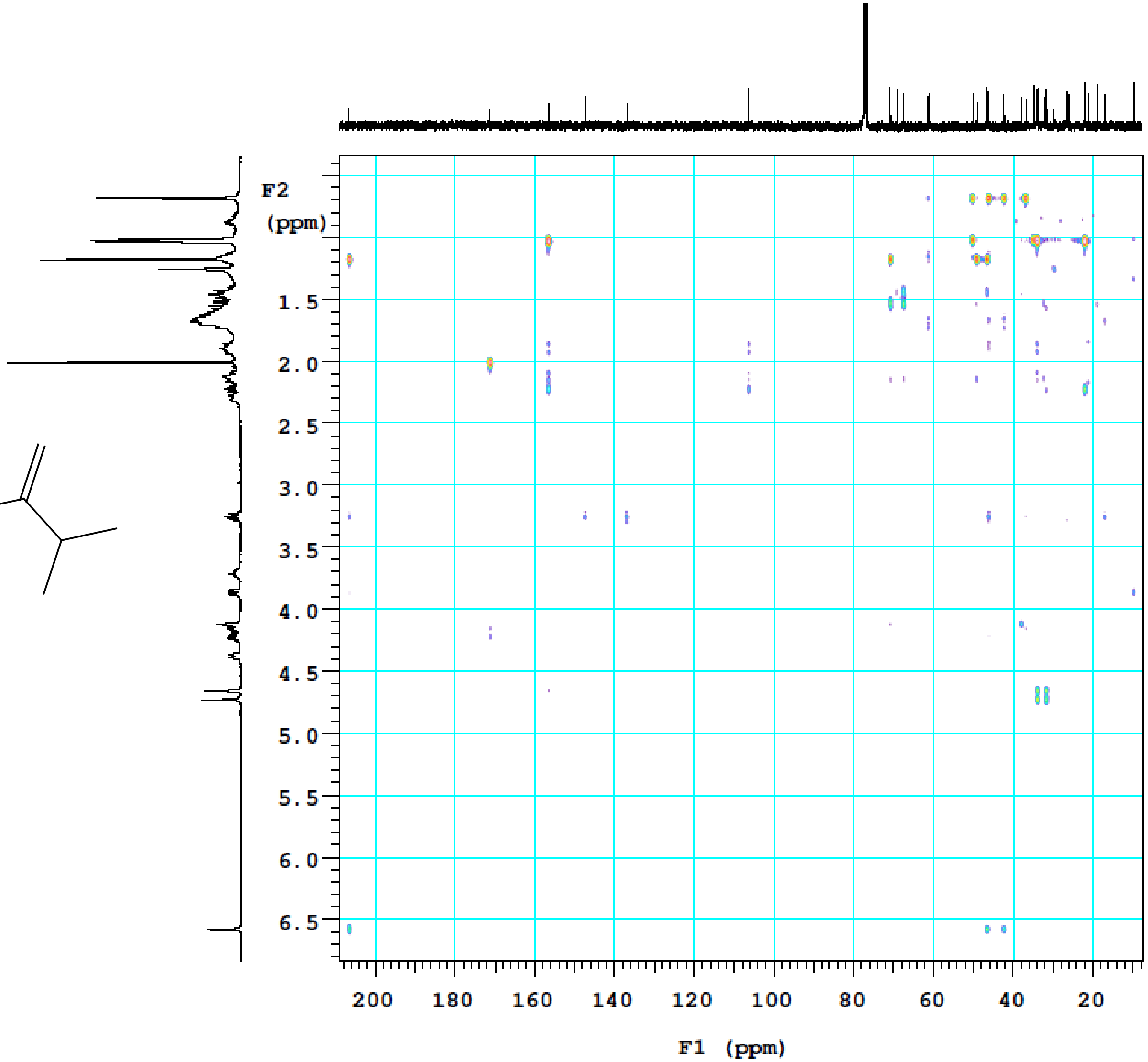

Figure S13. NOESY spectrum (400 MHz) of compound **2** in $CDCl_3$.

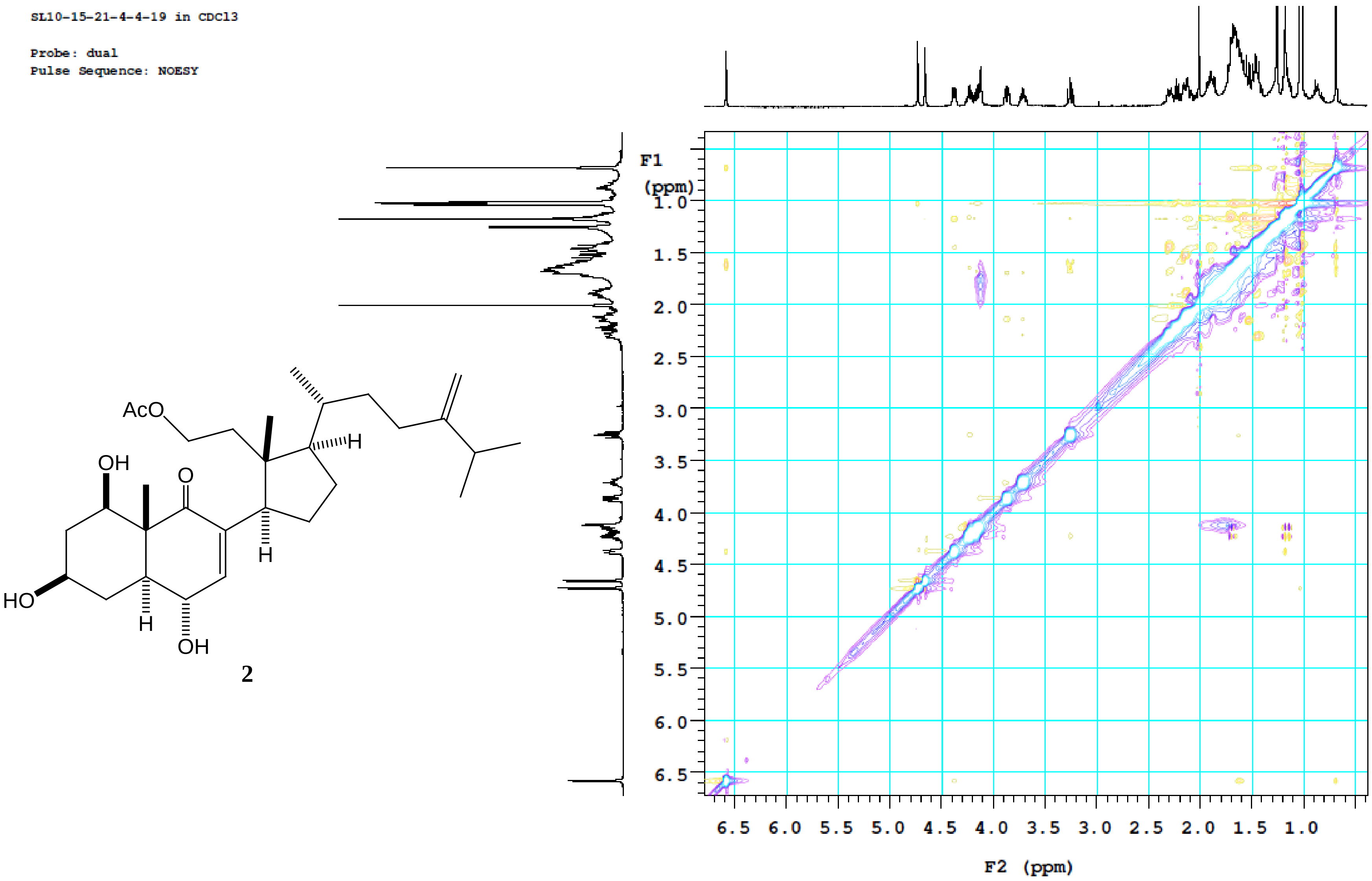

Supplement: Supplementary File 1 — Supplementary Materials (PDF, 610 KB) [file marinedrugs-11-03288-s001.pdf]
